# Integrative Pathway Analysis of SNP and Metabolite Data Using a Hierarchical Structural Component Model

**DOI:** 10.3389/fgene.2022.814412

**Published:** 2022-03-24

**Authors:** Taeyeong Jung, Youngae Jung, Min Kyong Moon, Oran Kwon, Geum-Sook Hwang, Taesung Park

**Affiliations:** ^1^ Interdisciplinary Program in Bioinformatics, Seoul National University, Seoul, South Korea; ^2^ Korea Integrated Metabolomics Research Group, Western Seoul Center, Korea Basic Science Institute, Seoul, South Korea; ^3^ Department of Internal Medicine, Seoul National University Boramae Medical Center, Seoul, South Korea; ^4^ Department of Nutritional Science and Food Management, Graduate Program in System Health Science and Engineering, Ewha Womans University, Seoul, South Korea; ^5^ Department of Statistics, Seoul National University, Seoul, South Korea

**Keywords:** pathway analysis, multi-omics integration, mGWAS, metabolite, SNP

## Abstract

Integrative multi-omics analysis has become a useful tool to understand molecular mechanisms and drug discovery for treatment. Especially, the couplings of genetics to metabolomics have been performed to identify the associations between SNP and metabolite. However, while the importance of integrative pathway analysis is increasing, there are few approaches to utilize pathway information to analyze phenotypes using SNP and metabolite. We propose an integrative pathway analysis of SNP and metabolite data using a hierarchical structural component model considering the structural relationships of SNPs, metabolites, pathways, and phenotypes. The proposed method utilizes genome-wide association studies on metabolites and constructs the genetic risk scores for metabolites referred to as genetic metabolomic scores. It is based on the hierarchical model using the genetic metabolomic scores and pathways. Furthermore, this method adopts a ridge penalty to consider the correlations between genetic metabolomic scores and between pathways. We apply our method to the SNP and metabolite data from the Korean population to identify pathways associated with type 2 diabetes (T2D). Through this application, we identified well-known pathways associated with T2D, demonstrating that this method adds biological insights into disease-related pathways using genetic predispositions of metabolites.

## 1 Introduction

The advances in biological techniques have led to the generation of multiple omics (multi-omics) data, which contribute to a better understanding of biological mechanisms and diseases. For instance, the next-generation sequencing (NGS) technology for genome-wide data and mass spectrometry for quantitative metabolic data allow us to generate multi-omics data from the same samples at a low cost (Metzker, 2010; Suhre and Gieger, 2012). These technical improvements have enabled multi-omics data analysis to become a useful tool in biomedical research.

Genome-wide association studies (GWAS) have been conducted worldwide to identify single nucleotide polymorphisms (SNPs) associated with various diseases or phenotypes.

An intermediate variable, linking genetic variants and phenotype, is suggested to consider the effects of genes and environmental factors in overcoming the limitation of GWAS (Kronenberg, 2012). One of the potential intermediate variables is serum metabolite concentration, providing a direct readout of biological processes, to connect genetic factors and diseases (Illig et al., 2010; Kronenberg, 2012). Recently, metabolite genome-wide association studies (mGWAS) and metabolic quantitative trait loci (mQTL) analyses have been conducted by utilizing SNP and metabolite data together (Zhang et al., 2017; Park et al., 2019; Ouyang et al., 2021). In addition, to explore the association between SNPs and metabolites, disease-related metabolomic markers using SNPs were investigated through Mendelian randomization (Moayyeri et al., 2018). Even though many studies attempted to analyze SNP and metabolite data together, most studies have mainly focused on either analyzing statistical associations between SNPs and metabolites or discovering metabolomic markers of phenotypes using SNPs.

Since pathway analysis can give a more intuitive interpretation of the biological system, several methods have been proposed for pathway analysis that focuses on identifying significant pathways related to certain traits of interest (García-Campos et al., 2015; Kao et al., 2017). Specifically, pathway analysis using multi-omics data has now become popularly used in recent bioinformatics research. While the importance of integrative pathway analysis is increasing, there have been few studies about integrating SNPs and metabolite data (Kao et al., 2017). In this study, we focus on integrative pathway analysis of SNPs and metabolite data.

Here, we propose an integrative pathway analysis of SNP and metabolite data using a hierarchical structural component model. This method calculates genetic risk scores of metabolites and investigates pathways associated with phenotypes through the genetic risk scores. This approach is based on our earlier work Pathway-based approach using HierArchical components of collapsed RAre variants Of High-throughput sequencing data (PHARAOH) (Lee et al., 2016). PHARAOH uses rare variants to construct collapsed genes and performs pathway analysis using these gene-summaries. PHARAOH simultaneously analyzes the entire collapsed genes and the entire pathways in a hierarchical model (Lee et al., 2016). We utilize this main framework of PHARAOH and mGWAS for the integration of SNP and metabolite data and refer to this method as a Hierarchical Structural Component Model of SNP and Metabolite data for pathway analysis (HisCoM-SM).

The genetic metabolomic score (GMS) is calculated by summing the effects of the corresponding SNPs on each metabolite and then is used for pathway analysis in PHARAOH. HisCoM-SM adopts the ridge penalties to both GMSs and pathways to identify pathways while controlling for potential correlations between GMSs and between pathways.

Here, we apply HisCoM-SM to SNP and metabolite data from Korean Association REsource (KARE) cohort to identify pathways associated with T2D. Note that T2D is a metabolic disorder that is affected by genetic factors and environmental exposure simultaneously (Murea et al., 2012). Through this application to the KARE dataset, we demonstrate that HisCoM-SM can identify previously reported pathways, including insulin secretion and insulin resistance, associated with T2D, using genetic predispositions of metabolites (Weyer et al., 2001; Dayeh et al., 2014; Kahn et al., 2014).

The HisCoM-SM is available at https://statgen.snu.ac.kr/software/HisCoM_SM.

## 2 Materials and Methods

### 2.1 SNP Data

The SNP data was generated by the Affymetrix Genome-Wide Human SNP array 5.0. from the Korea Association REsource (KARE) project. KARE is based on Ansan and Ansung Korean population cohort among 10,038 participants which was initiated in 2001 (Cho et al., 2009). This chip originally consisted of 8,840 individuals and 352,228 SNPs. We applied quality control to our SNP data to reduce the biases and used common variants for our analysis (Turner et al., 2011). For quality control of SNP data, the genotypes with over 0.1 missing rates and Hardy-Weinberg equilibrium p-values < $10^{-6}$ were excluded. To use only common variants, the genotypes with minor allele frequency (MAF) ≤  0.05 were excluded. Then, we retained the individuals who have metabolite data and whose calling rate >0.9. After quality control of SNPs from the KARE dataset using PLINK 1.90, a total of 312,116 SNPs were analyzed in this work (Chang et al., 2015).

### 2.2 Metabolite Data

The serum metabolites in the 691 participants were quantitatively analyzed by a targeted metabolomics approach using liquid chromatography-mass spectrometry (LC-MS). 64 metabolites were measured in this work. The metabolites of each subject were measured at the fifth follow-up in the KARE dataset. Among 64 metabolites, 53 were mapped to 101 pathways. The 53 metabolites were classified into eight categories. Table 1 shows the number of metabolites in each category. The list of metabolites and the eight categories of metabolites are shown in Supplementary Table S1. 627 samples were available with both SNPs and metabolite data. Among these samples, 309 samples are controls (normal) and 318 samples are cases (pre-T2D and T2D). For metabolite data, systematical error removal using random forest (SERRF) was used for batch effect correction to remove variation due to instrument and injection time (Fan et al., 2019).

**TABLE 1:** Number of metabolites in each category.

Category	Number of metabolites
Alkaloids and derivatives	1
Benzenoids	2
Lipids and lipid-like molecules	1
Nucleosides, nucleotides, and analogues	4
Organic acids and derivatives	33
Organic nitrogen compounds	4
Organic oxygen compounds	1
Organoheterocyclic compounds	7

### 2.3 Diagnosis of Type 2 Diabetes

The criteria for diagnosis of T2D are 1) fasting blood glucose (FBS) ≥  126 mg/dl, 2) 2-h postprandial glucose (2 PP) ≥  200 mg/dl, 3) HbA1c ≥  6.5 (%), and 4) treatment of drug. Pre-diabetic (preT2D) individuals are diagnosed by the criteria—1) 100 mg/dl ≤ FBS < 126 mg/dl, 2) 140 mg/dl ≤  2 PP <  200 mg/dl, 3) 5.6% < HbA1c < 6.5%, and 4) no treatment of drug. The criteria for normal individual are 1) FBS <100, 2) 2 PP < 140, 3) HbA1c ≤ 5.6% , and 4) no treatment of drug. Here, we regarded T2D and preT2D individuals as cases, and normal individuals as controls. The baseline characteristics of those samples are shown in Table 2.

**TABLE 2:** The characteristics of the subjects in each case (pre T2D + T2D) and control (Normal) group.

	Case	Control	p-value
Male	157 (49.37%)	157 (50.81%)	0.7794
Age (years)	58.26	57.32	0.0653
BMI	25.22	24.60	0.0059
Number of subjects	318	309	—

### 2.4 HisCoM-SM

The framework of HisCoM-SM consists of two steps. The step 1 is to calculate genetic risk scores of metabolite data referred to genetic metabolomic scores (GMSs). Genetic effects of metabolites are estimated and then used to calculate the GMSs. The step 2 is to perform pathway analysis using the calculated GMSs by step 1. To perform pathway analysis, a hierarchical structural component model (HisCoM) is used. HisCoM consists of three layers which are input layer, latent layer, and outcome layer. In our work, GMSs are used as input variables, pathways are used as latent variables, and binary phenotype is used as outcome variable. The two steps of the process are described in more detail below.

#### 2.4.1 Calculation of GMSs

To perform pathway analysis using SNP and metabolite data, we first construct the GMSs. Here, we used two methods for calculating GMSs. The first score was derived from the single-SNP association test for metabolites. To do that, we applied linear regression for each metabolite adjusted for age and sex and calculated the GMSs by clumping and thresholding to remove redundant correlated effects due to linkage disequilibrium (LD) using PLINK (Chang et al., 2015). Clumping is the process of selecting the most significant SNP iteratively, computing correlation between this SNP and nearby SNPs within a genetic distance of 250 k, and removing all the nearby SNPs with highly correlation ( $r^{2}>0.2)$ (Privé et al., 2019). Thresholding is the process of filtering out variants with p-values greater than a given threshold level (Privé et al., 2019). Then, the GMSs are calculated from the effects of remaining SNPs after clumping and thresholding using PLINK (Chang et al., 2015).

The second score is based on the genetic best linear unbiased prediction (GBLUP) method from Genome-wide Complex Trait Analysis (GCTA) software (Yang et al., 2011). All SNPs are treated as random effects in a mixed linear model adjusted for fixed effects of sex and age (Yang et al., 2011). In GBLUP, the effects of all SNPs are estimated by the genetic relationship matrix (GRM) representing the relatedness of individuals’ SNPs (Yang et al., 2011). The GRM is used to estimate the effects of all SNPs and only 20% of SNPs with a high absolute value of the effect are used to construct the GMSs. Then, the remaining SNP effects are used to construct GMSs using PLINK (Chang et al., 2015).

#### 2.4.2 Pathway Analysis Using GMSs in a Hierarchical Component Model

**FIGURE 1:**
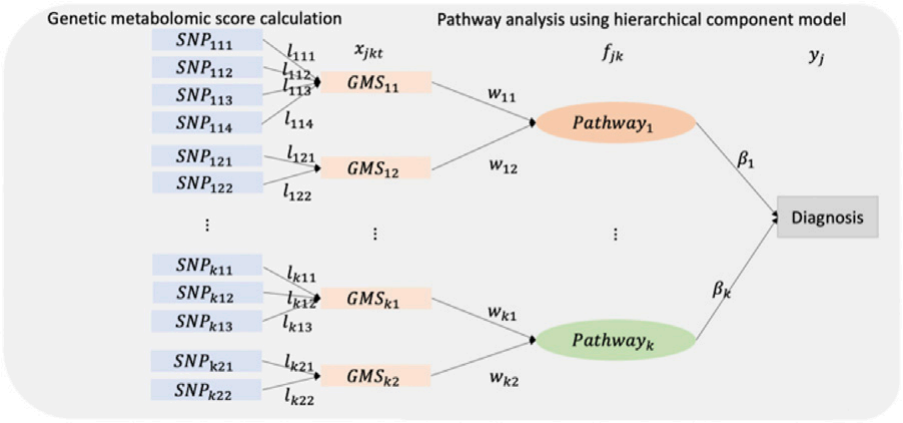
A schematic diagram of the HisCoM-SM.

After constructing the GMSs, pathway analysis is performed. Figure 1 shows the diagram of HisCoM-SM. For each metabolite, the correlated SNPs are selected by a single SNP association test and GBLUP. Then, GMSs are derived as a linear combination of these SNPs. Thus, each metabolite is linearly correlated with the selected multiple SNPs. Similarly, each pathway is also linearly correlated with multiple metabolites. First, an individual pathway is mapped to the metabolites using the KEGG pathway database. Next, the latent variables representing pathways are derived as a linear combination of these metabolites. Then, the binary outcome is used to estimate the effect of the relationship between pathways and the phenotype. Let $y_{j}$ be the binary outcome of the $j^{th}$ individual, K be the number of pathways, $T_{k\;}$ be the number of GMSs in the $k^{th}$ pathway. The $x_{jkt}$ denotes GMS which is a continuous value, the $w_{kt}$ represents weight for $x_{jkt}$ , and $\beta_{k}$ denotes the coefficient for pathway. Then, the proposed HisCoM model is defined as follows:

$$logit\left(\pi_{j}\right)=\;\beta_{0}+\sum_{k=1}^{K}\left[\sum_{t=1}^{T_{k}}w_{kt}x_{jkt}\right]\beta_{k}\;$$ (1)

To estimate the parameters of the model, we maximize a penalized log-likelihood function (Eq. 2) and use alternating least squares (ALS) for minimizing the objective function (Lee et al., 2016). Let $p\left(y_{j};\gamma_{j},\delta \right)$ be the probability distribution function for the phenotype $y_{j}$ , $\lambda_{M}$ and $\lambda_{P}$ denote ridge parameters added for potential multicollinearities between GMSs and between pathways, respectively. After determining the ridge parameters $\lambda_{M}$ and $\lambda_{P}$ by five-fold cross-validation, the coefficients $w_{kt}$ and $\beta_{k}$ are estimated by ALS algorithm. In ALS algorithm, $\beta_{k}$ are updated in a least square manner with $w_{kt}$ fixed. Likewise, $w_{kt}$ are updated with $\beta_{k}$ fixed. This ALS algorithm is iterated until convergence.

$$\phi =\sum_{j=1}^{N}\log p\left(y_{j};\gamma_{j},\delta \right)-1/2\lambda_{M}\sum_{k=1}^{K}\sum_{t=1}^{T_{k}}w_{kt}^{2}-1/2\lambda_{P}\;\sum_{k=0}^{K}\beta_{k}^{2}$$ (2)

After estimation, the phenotype is resampled 100,000 times through permutation to generate the null distribution of coefficients of pathways to calculate empirical p-values. To correct the multiple comparisons problem, the false discovery rate (FDR) is applied (Benjamini and Hochberg, 1995). Here, we use the WISARD (workbench for integrated superfast association studies for related datasets) to perform integrative pathway analysis using GMSs (Lee et al., 2018).

## 3 Results

### 3.1 Metabolite Genome-wide Association Study in KARE Dataset

**FIGURE 2:**
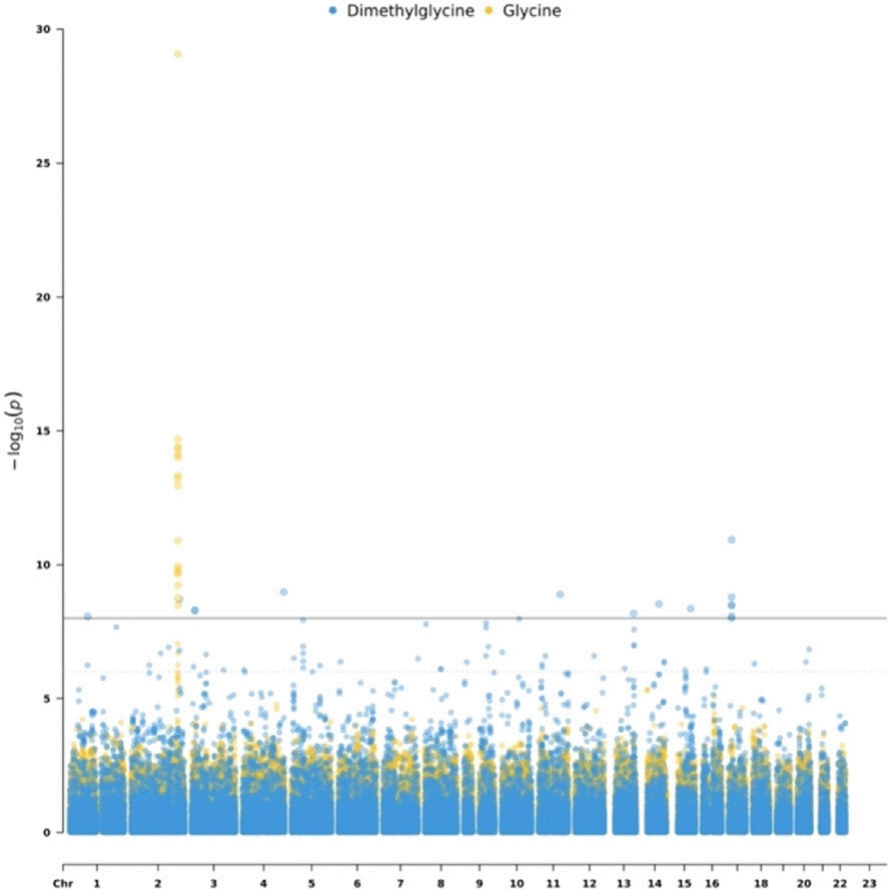
Manhattan plot for Dimethylglycine and Glycine

To detect genetic variants associated with metabolites, we performed the mGWAS using linear regression, adjusting for sex and age. Out of 53 metabolites, only two metabolites have significantly (p < 1e-8) associated SNPs. Specifically, we identified 17 SNPs associated with Glycine which is related to insulin sensitivity and secretion (Floegel et al., 2013). These SNPs are located on chromosome 2. We also identified 15 SNPs associated with Dimethylglycine. The list of the identified mQTL is shown in Supplementary Table S2. Figure 2 is a Manhattan plot for SNPs associated with Glycine and Dimethylglycine.

### 3.2 Pathway Analysis of T2D

HisCoM-SM was applied to SNP and metabolite data of T2D/preT2D patients and normal samples from the KARE dataset which is a large Korean population-based cohort. We first mapped the KEGG pathway database with metabolite data. Among 64 metabolites, 53 metabolites were mapped to 101 pathways. Then, the GMSs were used as components of pathways, which are latent variables in the model. Note that we used the two methods to construct GMSs: 1) Single-SNP association-based GMSs and 2) GBLUP-based GMSs. Those two methods are discussed in detail in the Methods section. The lists of identified pathways with HisCoM-SM based on single-SNP association denoted by HisCoM-SM (single) and HisCoM-SM based on GBLUP denoted by HisCoM-SM(GBLUP) are shown in Supplementary Tables S3, S4, respectively.

To detect the significant pathways associated with T2D in HisCoM-SM, we selected the pathways with high absolute coefficient values and low q-values. The metabolic pathway (map 01100) had the highest absolute effect value and the lowest q-value in both HisCoM-SM (single) and HisCoM-SM(GBLUP). Among the 49 metabolites contained in this pathway, Arginine, Tryptophan, Lactate, Trimethylamine N-oxide (TMAO), *Trans*-4-Hydroxy-L-proline, and Hippurate were significant in both HisCoM-SM methods. Arginine facilitates the action of glucose to stimulate insulin release (Gerich et al., 1974). In addition to Arginine, the other five metabolites have also been reported as risk factors for the incidence of T2D or the prevalence of T2D (Van Doorn et al., 2007; Crawford et al., 2010; Chen et al., 2016; Shan et al., 2017; Tang et al., 2017). In addition, both HisCoM-SM methods identified the same pathway with the second-highest absolute coefficient value and the lowest q-value. This pathway is the biosynthesis of amino acids (map 01230) and has also been reported to be associated with T2D in previous studies (Aichler et al., 2017; Lu et al., 2019). These results demonstrate that HisCoM-SM(single) and HisCoM-SM(GBLUP) can yield consistent results and identify pathways associated with T2D.

### 3.3 Comparison of HisCoM-SM to Conventional HisCoM Using Metabolite Data

**FIGURE 3:**
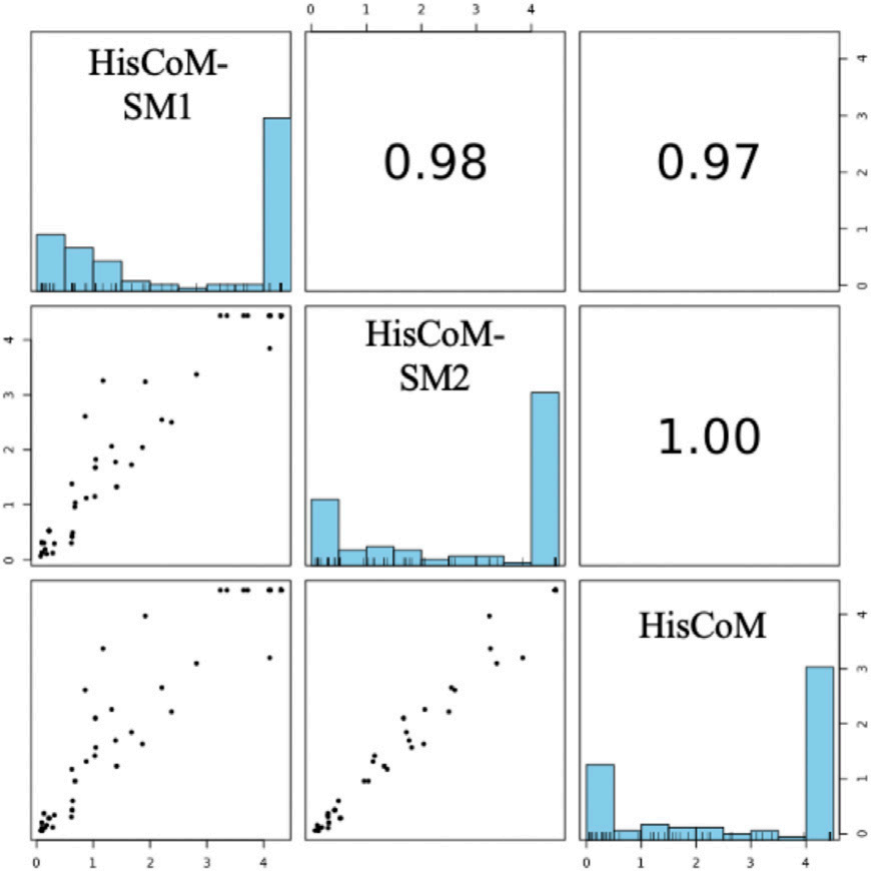
Pairwise scatter plot of 101 pathways’ FDR q-values calculated by HisCoM-SM methods and HisCoM using metabolite. Note that His-CoM-SM1 represents HisCoM-SM(single) and HisCoM-SM2 represents HisCoM-SM(GBLUP).

**FIGURE 4:**
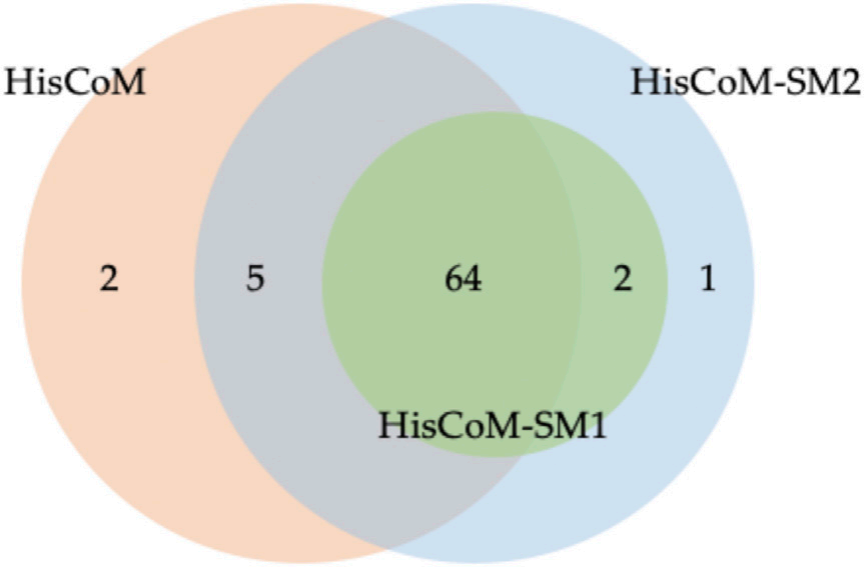
Venn Diagram for numbers of significant pathways detected for each method. Note that HisCoM-SM1 represents HisCoM-SM(single) and HisCoM-SM2 represents HisCoM-SM(GBLUP).

For comparison purposes, we applied the conventional HisCoM to KARE metabolite data to identify the T2D related pathways (Supplementary Table S5). Table 3 summarizes the commonly significant (FDR q-value < 0.05) pathways by HisCoM-SM(single), HisCoM-SM(GBLUP), and conventional HisCoM using only metabolite data. These commonly significant pathways are categorized by the KEGG pathway category and subcategory. Metabolism is the category that has the greatest number of significant pathways. Among the 64 significant pathways, 31 pathways are included in the metabolism category. Figure 3 is a scatter plot for the FDR q-values and the correlation coefficients for each pair of methods. Here, the q-values of HisCoM-SM and HisCoM showed quite consistent patterns yielding high correlation coefficients. Figure 4 is a Venn diagram to show the numbers of significant pathways (FDR q-value < 0.05) shared by different methods. Note that 64 out of 74 significant pathways were commonly identified by all three methods, indicating that HisCoM based methods yielded quite consistent results. Also, all pathways identified by HisCoM-SM (single) were identified by HisCoM-SM(GBLUP).

**TABLE 3:** Identified common pathways in HisCoM-SM and conventional HisCoM (q-value < 0.05). The pathways are categorized by KEGG pathway categories and KEGG pathway subcategories. The values in parenthesis are the number of pathways included in the KEGG pathway.

KEGG pathway category	KEGG pathway subcategory	Pathway
Cellular Processes (3)	Cell growth and death	Ferroptosis
	Cell motility	Regulation of actin cytoskeleton
	Cellular community - eukaryotes	Gap junction
Environmental Information Processing (4)	Membrane transport	ABC transporters
	Signal transduction	mTOR signaling pathway/Sphingolipid signaling pathway
	Signaling molecules and interaction	Neuroactive ligand-receptor interaction
Genetic Information Processing (2)	Folding, sorting, and degradation	Sulfur relay system
	Translation	Aminoacy-tRNA biosynthesis
Human Diseases (9)	Drug resistance: antineoplastic	Antifolate resistance
	Endocrine and metabolic disease	Insulin resistance
	Neurodegenerative disease	Amyotrophic lateral sclerosis/Parkinson disease
	Substance dependence	Alcoholism/Amphetamine addiction/cocaine addiction/Morphine addiction/Nicotine addiction
Metabolism (31)	Amino acid metabolism	Alanine, aspartate and glutamate metabolism/Arginine and proline metabolism/Arginine biosynthesis/Cysteine and methionine metabolism/Glycine, serine and threonine metabolism/Histidine metabolism/Phenylalanine metabolism/Phenylalanine, tyrosine and tryptophan biosynthesis/Tyrosine metabolism/Valine, leucine and isoleucine biosynthesis/Valine, leucine, and isoleucine degradation
	Biosynthesis of other secondary metabolites	Caffeine metabolism/Neomycin, kanamycin, and gentamicin biosynthesis
	Carbohydrate metabolism	Butanoate metabolism/Glyoxylate and dicarboxylate metabolism/Pyruvate metabolism
	Energy metabolism	Nitrogen metabolism
	Global overview maps	2-Oxocarboxylic acid metabolism/Biosynthesis of amino acids/Carbon metabolism/Metabolic pathways
	Metabolism of cofactors and vitamins	Nicotinate and nicotinamide metabolism/Pantothenate and CoA biosynthesis/Porphyrin and chlorophyll metabolism/Thiamine metabolism
	Metabolism of other amino acids	beta-Alanine metabolism/D-Arginine and D-ornithine metabolism/D-glutamine and D-glutamate metabolism/Glutathione metabolism/Taurine and hypotaurine metabolism
	Nucleotide metabolism	Purine metabolism
Organismal Systems (15)	Digestive system	Bile secretion/Mineral absorption/Pancreatic secretion/Protein digestion and absorption
	Endocrine system	Estrogen signaling pathway/Insulin secretion/Prolactin signaling pathway
	Excretory system	Proximal tubule bicarbonate reclamation
	Nervous system	Dopaminergic synapse/GABAergic synapse/Glutamatergic synapse/Long-term depression/Retrograde endocannabinoid signaling/Synaptic vesicle cycle
	Sensory system	Taste transduction

In Figure 4, conventional HisCoM identified two pathways that HisCoM-SM could not detect, one of which (selenocompound metabolism; map 00450) was previously reported to be associated with T2D (Shin et al., 2020). On the other hand, HisCoM-SM(GBLUP) identified three pathways, which conventional HisCoM could not find. Two out of these pathways were reported as associated with T2D. These two significant pathways are biotin metabolism (map 00780) and vascular smooth muscle contraction (map 04270) (Xie et al., 2006; Hashimoto et al., 2020). For biotin metabolism, several studies have shown that plasma triacylglycerol, low-density lipoprotein cholesterol (LDL), and fasting glucose are reduced in patients with T2D who take biotin supplementation (Maebashi et al., 1993; Revilla-Monsalve et al., 2006). Furthermore, biotin intake has been reported to be effective in improving glycaemic control through diabetic animal models (Reddi et al., 1988; Zhang et al., 1997).

## 4 Discussion

Several studies have suggested that pathway analysis using multi-omics data allows more insights into biological systems. Pathway analysis using more than one omics data is becoming increasingly common. However, few studies can identify disease-related pathways considering SNPs and metabolites together.

We proposed a novel pathway analysis integrating SNP and metabolite data. Our method introduced novel genetic metabolomic scores (GMSs) for pathway analysis. We used a single-SNP association and a GBLUP approach to construct GMSs. The calculated GMSs were used as components of pathways in a hierarchical model. The coefficients can be estimated by analyzing GMSs and pathways simultaneously, considering the correlations between these scores and between pathways, respectively.

We applied HisCoM-SM to the KARE cohort dataset. Our HisCoM-SM successfully identified pathways that were reported to be related to T2D. In our result, the pathways identified by HisCoM-SM and conventional HisCoM were almost overlapped, indicating that HisCoM-SM and HisCoM yielded quite consistent results, and the GMSs can be utilized for pathway analysis. Moreover, HisCoM-SM could identify the T2D-related pathways that the conventional HisCoM using only metabolite data could not detect. Since 53 targeted metabolomics in our analysis may cover only a small portion of the metabolome, modeling the effects of SNPs on these metabolites resulted in similar results from the conventional HisCoM method using only metabolites. We are planning to modify HisCoM-SM so that it allows for each pathway to have inputs from both genes and metabolites simultaneously. In other words, SNPs can directly contribute to pathways (not through metabolites), which also makes a more biological sense. The new model with a rewired structure is expected to improve the performance. We will leave it as a near-future study.

Here, we applied the clumping and thresholding process to generate genetic metabolomic scores using p-values from linear regression models. Instead of linear regression models, other approaches such as Kernel regression can be applied to detect non-linear relationships between SNPs and metabolites. Our HisCoM-SM can also use other GMSs such as the ones derived from the LD pred method (Vilhjálmsson et al., 2015). Also, once the effect of SNPs on each metabolite is obtained, it can be used to calculate the GMSs for other datasets only with SNPs. The GMSs can be calculated using the effects of SNPs. Regarding the estimation of effects of pathways and genetic metabolomic scores, we can use different types of penalty functions. For example, LASSO or Elastic Net can be easily incorporated into our model instead of the Ridge penalty. Furthermore, we can construct a predictive model using HisCoM-SM approach for diagnosis. Specifically, we will evaluate the prediction performance of HisCoM-SM and compare it with those of other models such as original HisCoM using only SNPs and metabolites in a near future.

We believe that our method may add practical biological insights into the disease-related pathways by genetic predispositions of metabolites and contribute to the understanding of molecular mechanisms and treatment for the disease.

## Data Availability

Publicly available datasets were analyzed in this study. This data can be found here: http://koreabiobank.re.kr.
