# Supplementary material for: Integrative Pathway Analysis of SNP and Metabolite Data Using a Hierarchical Structural Component Model
Source: Front Genet. 2022 Mar 24;13:814412. doi: 10.3389/fgene.2022.814412 (PMC8987531; doi:10.3389/fgene.2022.814412)
Supplement: Supplementary file 1 [file DataSheet1.docx]

Supplementary Material

# Supplementary Tables

**Supplementary Table 1.** Categories of metabolites

| **Category** | **Metabolite** |
| --- | --- |
| Alkaloids and derivatives | Trigonelline |
| Benzenoids | Hippurate, Norepinephrine |
| Lipids and lipid-like molecules | Acetylcarnitine |
| Nucleosides, nucleotides, and analogues | Inosine, S-adenosylhomocysteine, Uridine, Xanthosine |
| Organic acids and derivatives | Acetoacetate, Alanine, Arginine, Asparagine, Aspartate, Betaine, Citrulline, Creatine, Creatinine, Cystathionine, Cysteine, Dimethylglycine, Gamma Aminobutyrate, Glutamate, Glutamine, Glycine, Histidine, Isoleucine, Lactate, Leucine, Lysine, Methionine, Phenylalanine, Proline, Pyroglutamic acid, Serine, Succinate, Taurine, Threonine, Trans 4-Hydroxy-L-proline, Tyrosine, Urea, Valine |
| Organic nitrogen compounds | Acetylcholine, Carnitine, Choline, Trimethylamine-N-oxide |
| Organic oxygen compounds | Kynurenine |
| Organoheterocyclic compounds | Guanine, Hypoxanthine, Nicotinamide, Riboflavin, Tryptophan, Uric acid, Xanthine |

**Supplementary Table 2.** Significant mQTL from GWAS of metabolites used in this analysis (p-value < 1e-8)

| **Metabolite** | **SNP** | **Chr** | **Position** | **Gene** | **P-value** |
| --- | --- | --- | --- | --- | --- |
| Dimethylglycine (DMG) | rs11163293 | 1 | 81669843 | HNRNPA3P14, LPHN2 | 8.596E-09 |
|  | rs2618141 | 2 | 218044863 | DIRC3 | 1.949E-09 |
|  | rs4449299 | 3 | 14573969 | GRIP2-RHBDF1P1 | 5.23E-09 |
|  | rs13084053 | 3 | 14576078 |  | 4.965E-09 |
|  | rs1443903 | 4 | 186558996 | LRP2BP, ANKRD37, UFSP2 | 1.038E-09 |
|  | rs11219937 | 11 | 98819238 | CNTN5 | 1.281E-09 |
|  | rs2227869 | 13 | 102313086 | BIVM-ERCC5, ERCC5 | 6.548E-09 |
|  | rs2803956 | 14 | 72149217 | RP3-514A23.2-DPF3 | 2.919E-09 |
|  | rs2043692 | 15 | 78259298 | FAH | 4.343E-09 |
|  | rs11656730 | 17 | 13836690 | COX10-AS1 | 1.596E-09 |
|  | rs8077837 | 17 | 13886527 | COX10-AS1 | 1.158E-11 |
|  | rs17677105 | 17 | 13889230 | COX10-AS1 | 3.295E-09 |
|  | rs9901800 | 17 | 13945320 | COX10 | 9.982E-09 |
|  | rs1017077 | 17 | 13945797 | COX10 | 8.484E-09 |
|  | rs4486949 | 17 | 13946431 | COX10 | 3.221E-09 |
| Glycine | rs4142152 | 2 | 211254288 | CPS1, CPS1-RP11-212J19.1 | 1.147E-10 |
|  | rs4142151 | 2 | 211254358 | CPS1, CPS1-RP11-212J19.1 | 2.062E-10 |
|  | rs2371015 | 2 | 211257854 | CPS1-RP11-212J19.1 | 2.062E-10 |
|  | rs12470865 | 2 | 211266007 | CPS1-RP11-212J19.1 | 5.83E-10 |
|  | rs2371019 | 2 | 211266745 | CPS1-RP11-212J19.1 | 1.505E-10 |
|  | rs10204632 | 2 | 211282010 | CPS1-RP11-212J19.1 | 1.118E-13 |
|  | rs10180880 | 2 | 211282067 | CPS1-RP11-212J19.1 | 4.584E-14 |
|  | rs10205035 | 2 | 211282569 | CPS1-RP11-212J19.1 | 6.165E-14 |
|  | rs2287414 | 2 | 211289312 | CPS1-RP11-212J19.1 | 9.496E-15 |
|  | rs16844839 | 2 | 211289490 | CPS1-RP11-212J19.1 | 8.321E-30 |
|  | rs41517444 | 2 | 211317177 | CPS1-RP11-212J19.1 | 2.023E-15 |
|  | rs2111716 | 2 | 211331807 | CPS1-RP11-212J19.1 | 1.687E-09 |
|  | rs12622504 | 2 | 211338520 | CPS1-RP11-212J19.1 | 4.59E-15 |
|  | rs2111714 | 2 | 211342607 | CPS1-RP11-212J19.1 | 7.519E-15 |
|  | rs2887931 | 2 | 211343147 | CPS1-RP11-212J19.1 | 4.005E-15 |
|  | rs4673555 | 2 | 211346414 | CPS1-RP11-212J19.1 | 1.255E-11 |
|  | rs12478788 | 2 | 211389488 | CPS1-RP11-212J19.1 | 3.242E-09 |

**Supplementary Table 3.** Pathways identified by HisCoM-SM based on single-SNP association (q-value < 0.05)

| **Pathway** | **coefficient** | **p-value** | **q-value** |
| --- | --- | --- | --- |
| 2-Oxocarboxylic acid metabolism | 0.00051 | 2.00E-05 | 5.00E-05 |
| ABC transporters | 0.00065 | 2.00E-05 | 5.00E-05 |
| Alanine, aspartate, and glutamate metabolism | 0.00059 | 2.00E-05 | 5.00E-05 |
| Alcoholism | 0.00048 | 2.00E-05 | 5.00E-05 |
| Aminoacyl-tRNA biosynthesis | 0.00069 | 2.00E-05 | 5.00E-05 |
| Amphetamine addiction | 0.00048 | 2.00E-05 | 5.00E-05 |
| Amyotrophic lateral sclerosis | 0.00046 | 2.00E-05 | 5.00E-05 |
| Antifolate resistance | 0.00033 | 4.00E-05 | 8.00E-05 |
| Arginine and proline metabolism | 0.00049 | 2.00E-05 | 5.00E-05 |
| Arginine biosynthesis | 5.00E-04 | 2.00E-05 | 5.00E-05 |
| beta-Alanine metabolism | 0.00024 | 0.0262 | 0.04071 |
| Bile secretion | 0.00029 | 0.01306 | 0.02127 |
| Biosynthesis of amino acids | 0.00069 | 2.00E-05 | 5.00E-05 |
| Butanoate metabolism | 0.00043 | 2.00E-05 | 5.00E-05 |
| Caffeine metabolism | 0.00037 | 1.00E-04 | 0.00019 |
| Carbon metabolism | 6.00E-04 | 2.00E-05 | 5.00E-05 |
| Cocaine addiction | 0.00048 | 2.00E-05 | 5.00E-05 |
| Cysteine and methionine metabolism | 0.00051 | 2.00E-05 | 5.00E-05 |
| D-Arginine and D-ornithine metabolism | 0.00033 | 4.00E-05 | 8.00E-05 |
| D-glutamine and D-glutamate metabolism | 0.00044 | 2.00E-05 | 5.00E-05 |
| Dopaminergic synapse | 0.00033 | 4.00E-05 | 8.00E-05 |
| Estrogen signaling pathway | 0.00033 | 4.00E-05 | 8.00E-05 |
| Ferroptosis | 0.00046 | 2.00E-05 | 5.00E-05 |
| GABAergic synapse | 0.00044 | 2.00E-05 | 5.00E-05 |
| Gap junction | 0.00042 | 2.00E-05 | 5.00E-05 |
| Glutamatergic synapse | 0.00044 | 2.00E-05 | 5.00E-05 |
| Glutathione metabolism | 0.00046 | 2.00E-05 | 5.00E-05 |
| Glycine, serine and threonine metabolism | 4.00E-04 | 0.00724 | 0.01219 |
| Glyoxylate and dicarboxylate metabolism | 0.00046 | 2.00E-05 | 5.00E-05 |
| Histidine metabolism | 0.00045 | 2.00E-05 | 5.00E-05 |
| Insulin resistance | 0.00023 | 0.00362 | 0.0062 |
| Insulin secretion | 0.00042 | 2.00E-05 | 5.00E-05 |
| Long-term depression | 0.00042 | 2.00E-05 | 5.00E-05 |
| Metabolic pathways | 0.00081 | 2.00E-05 | 5.00E-05 |
| Mineral absorption | 0.00058 | 2.00E-05 | 5.00E-05 |
| Morphine addiction | 0.00042 | 2.00E-05 | 5.00E-05 |
| mTOR signaling pathway | 3.00E-04 | 0.00086 | 0.00152 |
| Neomycin, kanamycin and gentamicin biosynthesis | 0.00042 | 2.00E-05 | 5.00E-05 |
| Neuroactive ligand-receptor interaction | 0.00045 | 4.00E-05 | 8.00E-05 |
| Nicotinate and nicotinamide metabolism | 0.00027 | 0.03122 | 0.04778 |
| Nicotine addiction | 0.00043 | 2.00E-05 | 5.00E-05 |
| Nitrogen metabolism | 0.00044 | 2.00E-05 | 5.00E-05 |
| Pancreatic secretion | 0.00033 | 4.00E-05 | 8.00E-05 |
| Pantothenate and CoA biosynthesis | 0.00039 | 4.00E-05 | 8.00E-05 |
| Parkinson disease | 0.00042 | 2.00E-05 | 5.00E-05 |
| Phenylalanine metabolism | 0.00039 | 0.00012 | 0.00022 |
| Phenylalanine tyrosine and tryptophan biosynthesis | 0.00035 | 0.00024 | 0.00044 |
| Porphyrin and chlorophyll metabolism | 0.00045 | 2.00E-05 | 5.00E-05 |
| Prolactin signaling pathway | 0.00033 | 4.00E-05 | 8.00E-05 |
| Protein digestion and absorption | 0.00069 | 2.00E-05 | 5.00E-05 |
| Proximal tubule bicarbonate reclamation | 0.00044 | 2.00E-05 | 5.00E-05 |
| Purine metabolism | 5.00E-04 | 2.00E-05 | 5.00E-05 |
| Pyruvate metabolism | 0.00043 | 2.00E-05 | 5.00E-05 |
| Regulation of actin cytoskeleton | 0.00033 | 4.00E-05 | 8.00E-05 |
| Retrograde endocannabinoid signaling | 0.00042 | 2.00E-05 | 5.00E-05 |
| Sphingolipid signaling pathway | 0.00033 | 4.00E-05 | 8.00E-05 |
| Sulfur relay system | 0.00047 | 2.00E-05 | 5.00E-05 |
| Synaptic vesicle cycle | 0.00043 | 4.00E-05 | 8.00E-05 |
| Taste transduction | 0.00043 | 2.00E-05 | 5.00E-05 |
| Taurine and hypotaurine metabolism | 0.00059 | 2.00E-05 | 5.00E-05 |
| Thiamine metabolism | 0.00042 | 2.00E-05 | 5.00E-05 |
| Tyrosine metabolism | 0.00036 | 0.00032 | 0.00058 |
| Ubiquinone and other terpenoid quinone biosynthesis | 0.00018 | 0.02458 | 0.03904 |
| Valine, leucine, and isoleucine biosynthesis | 0.00032 | 0.0024 | 0.00418 |
| Valine, leucine, and isoleucine degradation | 0.00029 | 0.00822 | 0.01361 |
| Vascular smooth muscle contraction | 0.00018 | 0.02474 | 0.03904 |

**Supplementary Table 4.** Pathways identified by HisCoM-SM based on GBLUP (q-value < 0.05)

| **Pathway** | **coefficient** | **p-value** | **q-value** |
| --- | --- | --- | --- |
| 2-Oxocarboxylic acid metabolism | 0.00112 | 2.00E-05 | 4.00E-05 |
| ABC transporters | 0.00124 | 2.00E-05 | 4.00E-05 |
| African trypanosomiasis | 0.00043 | 0.0143 | 0.02093 |
| Alanine, aspartate, and glutamate metabolism | 0.00112 | 2.00E-05 | 4.00E-05 |
| Alcoholism | 0.00106 | 2.00E-05 | 4.00E-05 |
| Aminoacyl-tRNA biosynthesis | 0.00137 | 2.00E-05 | 4.00E-05 |
| Amphetamine addiction | 0.00106 | 2.00E-05 | 4.00E-05 |
| Amyotrophic lateral sclerosis | 0.001 | 2.00E-05 | 4.00E-05 |
| Antifolate resistance | 0.00077 | 2.00E-05 | 4.00E-05 |
| Arginine and proline metabolism | 0.00103 | 2.00E-05 | 4.00E-05 |
| Arginine biosynthesis | 0.00104 | 2.00E-05 | 4.00E-05 |
| beta-Alanine metabolism | 0.00048 | 0.0109 | 0.01668 |
| Bile secretion | 0.00052 | 0.01242 | 0.01872 |
| Biosynthesis of amino acids | 0.00137 | 2.00E-05 | 4.00E-05 |
| Biotin metabolism | 0.00039 | 0.02888 | 0.04167 |
| Butanoate metabolism | 0.00093 | 2.00E-05 | 4.00E-05 |
| Caffeine metabolism | 0.00077 | 2.00E-05 | 4.00E-05 |
| Carbon metabolism | 0.00114 | 2.00E-05 | 4.00E-05 |
| Cocaine addiction | 0.00106 | 2.00E-05 | 4.00E-05 |
| Cysteine and methionine metabolism | 0.00088 | 2.00E-05 | 4.00E-05 |
| D-Arginine and D-ornithine metabolism | 0.00077 | 2.00E-05 | 4.00E-05 |
| D-glutamine and D-glutamate metabolism | 0.00093 | 2.00E-05 | 4.00E-05 |
| Dopaminergic synapse | 0.00077 | 2.00E-05 | 4.00E-05 |
| Estrogen signaling pathway | 0.00077 | 2.00E-05 | 4.00E-05 |
| Ferroptosis | 0.00095 | 2.00E-05 | 4.00E-05 |
| GABAergic synapse | 0.00094 | 2.00E-05 | 4.00E-05 |
| Gap junction | 0.00093 | 2.00E-05 | 4.00E-05 |
| Glutamatergic synapse | 0.00093 | 2.00E-05 | 4.00E-05 |
| Glutathione metabolism | 0.001 | 2.00E-05 | 4.00E-05 |
| Glycine, serine and threonine metabolism | 0.00085 | 0.00034 | 0.00058 |
| Glyoxylate and dicarboxylate metabolism | 0.00102 | 2.00E-05 | 4.00E-05 |
| Histidine metabolism | 0.00095 | 2.00E-05 | 4.00E-05 |
| Insulin resistance | 0.00045 | 0.00172 | 0.00285 |
| Insulin secretion | 0.00093 | 2.00E-05 | 4.00E-05 |
| Long-term depression | 0.00093 | 2.00E-05 | 4.00E-05 |
| Lysine degradation | -6.00E-04 | 0.00146 | 0.00246 |
| Metabolic pathways | 0.00154 | 2.00E-05 | 4.00E-05 |
| Mineral absorption | 0.00109 | 2.00E-05 | 4.00E-05 |
| Morphine addiction | 0.00093 | 2.00E-05 | 4.00E-05 |
| mTOR signaling pathway | 6.00E-04 | 0.00024 | 0.00043 |
| Neomycin, kanamycin and gentamicin biosynthesis | 0.00093 | 2.00E-05 | 4.00E-05 |
| Neuroactive ligand-receptor interaction | 0.00099 | 2.00E-05 | 4.00E-05 |
| Nicotinate and nicotinamide metabolism | 0.00059 | 0.0054 | 0.00866 |
| Nicotine addiction | 0.00093 | 2.00E-05 | 4.00E-05 |
| Nitrogen metabolism | 0.00093 | 2.00E-05 | 4.00E-05 |
| Pancreatic secretion | 0.00077 | 2.00E-05 | 4.00E-05 |
| Pantothenate and CoA biosynthesis | 0.00065 | 8.00E-05 | 0.00014 |
| Parkinson disease | 0.00093 | 2.00E-05 | 4.00E-05 |
| Phenylalanine metabolism | 0.00085 | 2.00E-05 | 4.00E-05 |
| Phenylalanine, tyrosine and tryptophan biosynthesis | 0.00082 | 2.00E-05 | 4.00E-05 |
| Porphyrin and chlorophyll metabolism | 0.00099 | 2.00E-05 | 4.00E-05 |
| Primary bile acid biosynthesis | -0.00057 | 0.00032 | 0.00056 |
| Prolactin signaling pathway | 0.00077 | 2.00E-05 | 4.00E-05 |
| Protein digestion and absorption | 0.00137 | 2.00E-05 | 4.00E-05 |
| Proximal tubule bicarbonate reclamation | 0.00093 | 2.00E-05 | 4.00E-05 |
| Purine metabolism | 0.00104 | 2.00E-05 | 4.00E-05 |
| Pyrimidine metabolism | 5.00E-04 | 0.00964 | 0.01498 |
| Pyruvate metabolism | 0.00078 | 2.00E-05 | 4.00E-05 |
| Regulation of actin cytoskeleton | 0.00077 | 2.00E-05 | 4.00E-05 |
| Retrograde endocannabinoid signaling | 0.00093 | 2.00E-05 | 4.00E-05 |
| Sphingolipid signaling pathway | 0.00077 | 2.00E-05 | 4.00E-05 |
| Sulfur relay system | 0.00075 | 2.00E-05 | 4.00E-05 |
| Synaptic vesicle cycle | 0.00098 | 2.00E-05 | 4.00E-05 |
| Taste transduction | 0.00093 | 2.00E-05 | 4.00E-05 |
| Taurine and hypotaurine metabolism | 0.00109 | 2.00E-05 | 4.00E-05 |
| Thiamine metabolism | 0.00095 | 2.00E-05 | 4.00E-05 |
| Tryptophan metabolism | 0.00043 | 0.0142 | 0.02093 |
| Tyrosine metabolism | 0.00083 | 2.00E-05 | 4.00E-05 |
| Ubiquinone and other terpenoid quinone biosynthesis | 0.00031 | 0.03348 | 0.04705 |
| Valine, leucine, and isoleucine biosynthesis | 6.00E-04 | 0.00194 | 0.00316 |
| Valine, leucine, and isoleucine degradation | 0.00055 | 0.00572 | 0.00903 |
| Vascular smooth muscle contraction | 0.00031 | 0.03354 | 0.04705 |

**Supplementary Table 5.** Pathways identified by HisCoM using metabolite data (q-value < 0.05)

| **Pathway** | **coefficient** | **p-value** | **q-value** |
| --- | --- | --- | --- |
| 2-Oxocarboxylic acid metabolism | 6.00E-04 | 2.00E-05 | 4.00E-05 |
| ABC transporters | 0.00067 | 2.00E-05 | 4.00E-05 |
| African trypanosomiasis | 0.00026 | 0.00504 | 0.00786 |
| Alanine, aspartate, and glutamate metabolism | 0.00061 | 2.00E-05 | 4.00E-05 |
| Alcoholism | 0.00056 | 2.00E-05 | 4.00E-05 |
| Aminoacyl-tRNA biosynthesis | 0.00074 | 2.00E-05 | 4.00E-05 |
| Amphetamine addiction | 0.00056 | 2.00E-05 | 4.00E-05 |
| Amyotrophic lateral sclerosis | 0.00053 | 2.00E-05 | 4.00E-05 |
| Antifolate resistance | 0.00041 | 2.00E-05 | 4.00E-05 |
| Arginine and proline metabolism | 0.00055 | 2.00E-05 | 4.00E-05 |
| Arginine biosynthesis | 0.00056 | 2.00E-05 | 4.00E-05 |
| beta-Alanine metabolism | 0.00026 | 0.0133 | 0.02005 |
| Bile secretion | 0.00029 | 0.00928 | 0.0142 |
| Biosynthesis of amino acids | 0.00074 | 2.00E-05 | 4.00E-05 |
| Butanoate metabolism | 5.00E-04 | 2.00E-05 | 4.00E-05 |
| Caffeine metabolism | 0.00041 | 2.00E-05 | 4.00E-05 |
| Carbon metabolism | 0.00062 | 2.00E-05 | 4.00E-05 |
| Cocaine addiction | 0.00056 | 2.00E-05 | 4.00E-05 |
| Cysteine and methionine metabolism | 5.00E-04 | 2.00E-05 | 4.00E-05 |
| D-Arginine and D-ornithine metabolism | 0.00041 | 2.00E-05 | 4.00E-05 |
| D-glutamine and D-glutamate metabolism | 5.00E-04 | 2.00E-05 | 4.00E-05 |
| Dopaminergic synapse | 0.00041 | 2.00E-05 | 4.00E-05 |
| Estrogen signaling pathway | 0.00041 | 2.00E-05 | 4.00E-05 |
| Ferroptosis | 0.00051 | 2.00E-05 | 4.00E-05 |
| GABAergic synapse | 0.00051 | 2.00E-05 | 4.00E-05 |
| Gap junction | 5.00E-04 | 2.00E-05 | 4.00E-05 |
| Glutamatergic synapse | 5.00E-04 | 2.00E-05 | 4.00E-05 |
| Glutathione metabolism | 0.00054 | 2.00E-05 | 4.00E-05 |
| Glycine, serine, and threonine metabolism | 5.00E-04 | 6.00E-05 | 0.00011 |
| Glyoxylate and dicarboxylate metabolism | 0.00056 | 2.00E-05 | 4.00E-05 |
| Histidine metabolism | 5.00E-04 | 2.00E-05 | 4.00E-05 |
| Insulin resistance | 0.00025 | 0.0013 | 0.00219 |
| Insulin secretion | 0.00049 | 2.00E-05 | 4.00E-05 |
| Long-term depression | 0.00049 | 2.00E-05 | 4.00E-05 |
| Lysine degradation | 0.00033 | 0.00146 | 0.00242 |
| Metabolic pathways | 0.00084 | 2.00E-05 | 4.00E-05 |
| Mineral absorption | 6.00E-04 | 2.00E-05 | 4.00E-05 |
| Morphine addiction | 0.00049 | 2.00E-05 | 4.00E-05 |
| mTOR signaling pathway | 3.00E-04 | 0.00046 | 0.00079 |
| Neomycin, kanamycin and gentamicin biosynthesis | 0.00049 | 2.00E-05 | 4.00E-05 |
| Neuroactive ligand-receptor interaction | 0.00054 | 2.00E-05 | 4.00E-05 |
| Nicotinate and nicotinamide metabolism | 0.00033 | 0.00334 | 0.00544 |
| Nicotine addiction | 5.00E-04 | 2.00E-05 | 4.00E-05 |
| Nitrogen metabolism | 5.00E-04 | 2.00E-05 | 4.00E-05 |
| Pancreatic secretion | 0.00041 | 2.00E-05 | 4.00E-05 |
| Pantothenate and CoA biosynthesis | 0.00035 | 0.00036 | 0.00063 |
| Parkinson disease | 0.00049 | 2.00E-05 | 4.00E-05 |
| Phenylalanine metabolism | -0.00044 | 2.00E-05 | 4.00E-05 |
| Phenylalanine, tyrosine and tryptophan biosynthesis | 0.00043 | 2.00E-05 | 4.00E-05 |
| Porphyrin and chlorophyll metabolism | 0.00054 | 2.00E-05 | 4.00E-05 |
| Primary bile acid biosynthesis | -0.00033 | 0.00024 | 0.00043 |
| Prolactin signaling pathway | 0.00041 | 2.00E-05 | 4.00E-05 |
| Protein digestion and absorption | 0.00074 | 2.00E-05 | 4.00E-05 |
| Proximal tubule bicarbonate reclamation | 5.00E-04 | 2.00E-05 | 4.00E-05 |
| Purine metabolism | 0.00056 | 2.00E-05 | 4.00E-05 |
| Pyrimidine metabolism | 0.00025 | 0.01834 | 0.02685 |
| Pyruvate metabolism | 0.00041 | 2.00E-05 | 4.00E-05 |
| Regulation of actin cytoskeleton | 0.00041 | 2.00E-05 | 4.00E-05 |
| Retrograde endocannabinoid signaling | 5.00E-04 | 2.00E-05 | 4.00E-05 |
| Selenocompound metabolism | -0.00017 | 0.03378 | 0.04805 |
| Sphingolipid signaling pathway | 0.00041 | 2.00E-05 | 4.00E-05 |
| Sulfur metabolism | 0.00026 | 0.02636 | 0.03803 |
| Sulfur relay system | 0.00041 | 2.00E-05 | 4.00E-05 |
| Synaptic vesicle cycle | 0.00053 | 2.00E-05 | 4.00E-05 |
| Taste transduction | 5.00E-04 | 2.00E-05 | 4.00E-05 |
| Taurine and hypotaurine metabolism | 0.00059 | 2.00E-05 | 4.00E-05 |
| Thiamine metabolism | 0.00051 | 2.00E-05 | 4.00E-05 |
| Tryptophan metabolism | 0.00026 | 0.00506 | 0.00786 |
| Tyrosine metabolism | 0.00044 | 2.00E-05 | 4.00E-05 |
| Valine, leucine, and isoleucine biosynthesis | 0.00032 | 0.00376 | 0.00603 |
| Valine, leucine, and isoleucine degradation | 0.00028 | 0.01564 | 0.02323 |
